# T16. GLUTAMATERGIC CHANGES IN UHR

**DOI:** 10.1093/schbul/sby016.292

**Published:** 2018-04-01

**Authors:** Christina Wenneberg, Brian Broberg, Egill Rostrup, Louise Birkedal Glenthøj, Birte Glenthoj, Merete Nordentoft, Tina Dam Kristensen

**Affiliations:** 1 Psychiatric Centre Copenhagen; 2 Center for Neuropsychiatric Schizophrenia Research; 3 Center for Neuropsychiatric Schizophrenia Research, Center for Clinical Intervention and Neuropsychiatric Schizophrenia Research, Psychiatric Center Glostrup, University of Copenhagen, Glostrup University Hospital; 4 Mental Health Centre Copenhagen; 5 Center for Neuropsychiatric Schizophrenia Research, Center for Clinical Intervention and Neuropsychiatric Schizophrenia Research, University of Copenhagen

## Abstract

**Background:**

The search for biomarkers may prove significant for short-term identification of UHR individuals (remission/non-remission). On a long-term basis, biomarkers might give the opportunity to delay or prevent psychotic episodes. Disturbances of the neurotransmitters glutamate and GABA have long been suspected to be involved in the pathophysiology of psychosis. These disorders have also found been found in people at UHR, making it a promising area for early detection.

Cognitive deficits in schizophrenia are present prior to the onset of psychosis, and may be linked to perturbed glutamate and GABA function. Data suggest that this link is already present in UHR states.

**Methods:**

Participants: UHR individuals who meet the CAARMS criteria recruited from Mental Health Services in the Capital Region of Denmark and matching healthy controls.

**Examinations:**

1H-MRS of the ACC and thalamus. Diagnostic and psychopathological tests: CAARMS, SCID, SOFAS, PSP, Cornblatt, SANS, BPRS, MADRS, YMRS, CGI, PAS, SPI-A, AQoL Cognitive tests as part of collaborative studies.

**Results:**

So far 116 UHR individuals and 42 healthy controls have been scanned (December 2017) Very early preliminary analysis of the baseline data finds no significant difference in glutamate levels (in ACC and thalamus) in UHR patients compared to matched healthy controls. Baseline data remains to be analysed in relation to relevant subgroups of patients e.g. based on clinical outcome. GABA analysis and analysis of follow-up data are also yet to be performed. Data will be ready for the meeting, and will be presented.

**Discussion:**

More studies are needed in this field, since results so far have been diverging.

Baseline data remains to be analysed in relation to relevant subgroups of patients e.g. based on clinical outcome. GABA analysis and analysis of follow-up data are also yet to be performed. Glutamate data will be presented at the meeting.

